# Male Circumcision and STI Acquisition in Britain: Evidence from a National Probability Sample Survey

**DOI:** 10.1371/journal.pone.0130396

**Published:** 2015-06-17

**Authors:** Virginia Homfray, Clare Tanton, Robert F. Miller, Simon Beddows, Nigel Field, Pam Sonnenberg, Kaye Wellings, Kavita Panwar, Anne M. Johnson, Catherine H. Mercer

**Affiliations:** 1 Research Department of Infection and Population Health, University College London, London, United Kingdom; 2 Virus Reference Department, Public Health England, London, United Kingdom; 3 Centre for Sexual and Reproductive Health Research, London School of Hygiene and Tropical Medicine, London, United Kingdom; IPO, Portuguese Institute of Oncology of Porto, PORTUGAL

## Abstract

**Background:**

It is well-established that male circumcision reduces acquisition of HIV, herpes simplex virus 2, chancroid, and syphilis. However, the effect on the acquisition of non-ulcerative sexually transmitted infections (STIs) remains unclear. We examined the relationship between circumcision and biological measures of three STIs: human papillomavirus (HPV), *Chlamydia trachomatis* and *Mycoplasma genitalium*.

**Methods:**

A probability sample survey of 15,162 men and women aged 16-74 years (including 4,060 men aged 16-44 years) was carried out in Britain between 2010 and 2012. Participants completed a computer-assisted personal interview, including a computer-assisted self-interview, which asked about experience of STI diagnoses, and circumcision. Additionally, 1,850 urine samples from sexually-experienced men aged 16-44 years were collected and tested for STIs. Multivariable logistic regression was used to calculate adjusted odds ratios (AOR) to quantify associations between circumcision and i) self-reporting any STI diagnosis and ii) presence of STIs in urine, in men aged 16-44 years, adjusting for key socio-demographic and sexual behavioural factors.

**Results:**

The prevalence of circumcision in sexually-experienced men aged 16-44 years was 17.4% (95%CI 16.0-19.0). There was no association between circumcision and reporting any previous STI diagnoses, and specifically previous chlamydia or genital warts. However, circumcised men were less likely to have any HPV type (AOR 0.26, 95% confidence interval (CI) 0.13-0.50) including high-risk HPV types (HPV-16, 18, 31, 33, 35, 39, 45, 51, 52, 56, 58, 59 and/or 68) (AOR 0.14, 95% CI 0.05-0.40) detected in urine.

**Conclusions:**

Circumcised men had reduced odds of HPV detection in urine. These findings have implications for improving the precision of models of STI transmission in populations with different circumcision prevalence and in designing interventions to reduce STI acquisition.

## Introduction

It is well-established that male circumcision reduces HIV acquisition.[1–3] A meta-analysis concluded that male circumcision is also associated with a lower risk of infection with herpes simplex virus type 2, chancroid and syphilis.[4] However, the effect of male circumcision on non-ulcerative sexually transmitted infections (STIs) remains unclear.

Human Papillomavirus (HPV) is highly prevalent.[5] Persistent infection with ‘high-risk’ types (HR-HPV) is essential for the development of cervical cancer in women, and anal cancer in both men and women. HR-HPV has also been associated with penile cancers in men.[6] ‘Possible HR-HPV’ types are those with no clear evidence of carcinogenicity in humans, and types HPV-6 and HPV-11 are responsible for around 85% of genital warts.[7] A meta-analysis using detection of HPV DNA and/or genital warts (by self-report/clinical examination) as outcomes found that circumcision was strongly protective against HPV DNA detection (summary odds ratio (OR) 0.57; 95% Confidence Interval (CI) 0.45–0.71), but with substantial between-study heterogeneity, which may partly reflect the fact that most of the data were from convenience samples with relatively small sample sizes, while circumcision did not afford protection against genital warts.[8]

*Chlamydia trachomatis* and *Mycoplasma genitalium* are bacterial STIs possibly associated with upper reproductive tract infection in women. A randomised trial of circumcision to prevent acquisition of HIV in Kenya found Chlamydia infection was reduced in the intervention arm, but this lacked significance (incidence rate ratio 0.87, 95% CI 0.65–1.16).[9] However, in the same trial, circumcision almost halved the odds of detecting *M*. *genitalium* (OR 0.54; 95% CI 0.29–0.99), after controlling for behavioural risks.[9] A similar trial in South Africa demonstrated a decrease in prevalence of *C*. *trachomatis* in circumcised participants (adjusted OR 0.56; 95% CI 0.32–1.00).[10] However, few other studies have investigated the association between circumcision and these pathogens.

Our study examined the relationship between male circumcision and reporting previous STI diagnoses or detecting HPV, *C*. *trachomatis* and *M*. *genitalium* in urine among men in the British general population.

## Materials and Methods

The third National Survey of Sexual Attitudes and Lifestyles (Natsal-3) was a stratified probability sample survey of 15,162 men and women (6293 men) aged 16–74 years in Britain, who were interviewed during 2010–2012. The overall response rate was 57.7%. Participants were interviewed using computer-assisted face-to-face and self-interviews (CASI). The CASI included questions about male circumcision, sexual behaviour and history of STI diagnoses. Further methodological details have been described elsewhere.[11,12]

After the interview, a sample of participants aged 16–44 years who reported at least one lifetime sexual partner were invited to provide urine for STI testing.[5,11] Samples were tested for HPV, *C*. *trachomatis* and *M*. *genitalium*. STI test results were available from 1,850 men. HPV types were classified as high-risk, possible high-risk or HPV-6/11 using the IARC Monograph Working Group classification [13] (see Table 2). Details of the urine collection methods and testing procedures are published elsewhere.[5] Briefly, detection of *C*. *trachomatis* used the Aptima Combo 2 assay (Hologic Gen-Probe) as the initial screen and all positive or equivocal results were confirmed with the Aptima monospecific assay. An in-house Luminex-based genotyping assay was used to detect HPV types.[14] *M*. *genitalium* detection used an in-house Real-Time PCR assay which targets the adhesin protein (MgPa) gene, [15] with positive or equivocal results confirmed using a Genprobe Mycoplasma test. [16]

Stata (version 12.1) was used for all statistical analyses accounting for the stratification, clustering, and weighting of the sample, which was broadly representative of the British population.[11] Analyses were additionally weighted for unequal urine selection probabilities and differential urine sample response.[11] The denominator was limited to men aged 16–44 years reporting one or more lifetime partner. Prevalence estimates of self-reported circumcision with 95% confidence intervals (CIs) are presented. Associations between circumcision and demographic and behavioural variables were examined using logistic regression to calculate odds ratios (ORs). Crude and age-adjusted ORs are presented. To examine the association between circumcision and self-reported STI diagnoses or biological outcomes multivariable analysis was used to calculate ORs adjusted for age, ethnicity, same-sex experience and either number of lifetime partners (for STI diagnosis outcomes) or number of partners without a condom in the past year (for biological outcomes) (adjusted OR [AOR]).

All participants were given an information leaflet to read prior to participating in the survey and had the opportunity to discuss this with the interviewer. Verbal informed consent was obtained for participation in the interview and interviewers had to confirm that respondents had read the information leaflet in the computer programme before commencing the interview. Participants gave written informed consent for anonymised testing of urine samples, without the return of results, the ethical rationale for which has been previously described.[14] The Natsal-3 study, including the consent procedures, was approved by the Oxfordshire Research Ethics Committee A (reference: 09/H0604/27). All participants provided their own consent to participate, however for 16–17 year olds living at home, a parent/guardian provided additional verbal assent for participation. At the time of writing, the Natsal-3 data are being prepared for archiving with the UK Data Archive in June 2015, but before then, researchers may contact the Natsal-3 team to seek advance access to the corresponding data, and are directed to the Natsal website for further information (www.natsal.ac.uk).

## Results

The prevalence of reported circumcision in sexually-experienced men aged 16–44 resident in Britain was 17.4% (95% CI 16.0–19.0). Prevalence increased with age, from 13.2% (95% CI 11.3–15.5) among men aged 16–24 to 18.4% (95% CI 15.5–21.6) among men aged 35–44 (Table 1). Prevalence of male circumcision varied greatly according to ethnicity and religion, with men from ethnic minority backgrounds being significantly more likely to report being circumcised than men of White ethnicity. In unadjusted analyses, the odds of circumcision were 45.01 (95%CI 24.26–83.51) times higher in Muslim men than in those of no religious background. This association remained after adjusting for age. There were no associations between circumcision and the key sexual behaviours studied.

**Table 1:** Variations in the prevalence of circumcision by key sociodemographic factors and sexual behaviours reported by sexually–experienced^1^ men aged 16–44 years in Britain’s third National Survey of Sexual Attitudes and Lifestyles (Natsal-3).

	Circumcised		OR	(95%CI)	p-value	Age-adjusted OR	(95%CI)		Denom. (unwt, wt)^1^
	%	(95%CI)							
**All**									
***Sociodemographics***									
**Age (years)**					p = 0.0005				
16–24	13.20%	(11.3–15.5)	1	-					*1343*, *979*
25–34	19.60%	(17.5–22.0)	1.6	(1.26–2.03)					*1425*, *1273*
35–44	18.40%	(15.5–21.6)	1.47	(1.12–1.94)					*777*, *1374*
**Relationship status**					p = 0.0324			p = 0.2641	
Living with partner	19.10%	(17.0–21.5)	1	-		1	-		*1603*, *2130*
Steady relationship, not cohabiting	13.80%	(11.0–17.2)	0.68	(0.50–0.91)		0.75	(0.54–1.05)		*769*, *586*
No steady relationship, previously cohabited	15.60%	(11.7–20.6)	0.78	(0.54–1.13)		0.8	(0.55–1.15)		*338*, *272*
No steady relationship, never cohabited	15.50%	(12.8–18.7)	0.78	(0.59–1.02)		0.88	(0.64–1.21)		*821*, *627*
**Academic qualifications** ^**2**^					p = 0.0012			p = 0.0003	
No academic qualifications	16.60%	(12.0–22.6)	1	-		1	-		*316*, *347*
Academic qualifications typically gained at age 16	13.60%	(11.5–16.1)	0.79	(0.51–1.21)		0.81	(0.53–1.25)		*1216*, *1248*
Studying for/attained further academic qualifications	19.80%	(17.7–22.1)	1.24	(0.83–1.86)		1.35	(0.90–2.04)		*1815*, *1860*
**Ethnic group**					p<0.0001			p<0.0001	
White	12.00%	(10.8–13.4)	1	-		1	-		*3106*, *3093*
Mixed	37.40%	(26.1–50.3)	4.38	(2.58–7.45)		4.63	(2.72–7.88)		*88*, *82*
Asian/Asian British	48.80%	(40.0–57.6)	6.97	(4.80–10.13)		6.83	(4.67–9.97)		*175*, *254*
Black/Black British	64.40%	(53.9–73.8)	13.26	(8.38–20.99)		13.27	(8.37–21.02)		*123*, *137*
Other	29.80%	(17.0–46.8)	3.10	(1.50–6.43)		3.15	(1.50–6.64)		*47*, *53*
**Religion**					p<0.0001			p<0.0001	
None	13.60%	(12.0–15.4)	1	-		1	-		*2288*, *2249*
Christian	16.20%	(13.6–19.3)	1.23	(0.96–1.58)		1.2	(0.93–1.55)		*1022*, *1091*
Muslim	87.60%	(79.5–92.8)	45.01	(24.26–83.51)		45.44	(24.62–83.86)		*119*, *149*
Hindu	3.90%	(1.2–11.7)	0.26	(0.08–0.83)		0.24	(0.07–0.79)		*45*, *69*
Other	21.50%	(13.3–32.9)	1.74	(0.96–3.16)		1.8	(0.98–3.27)		*66*, *63*
***Sexual behaviours***									
**Number of sexual partners** ^**3**^ **over the lifetime**					p = 0.256			p = 0.1224	
1	21.00%	(16.8–25.9)	1	-		1	-		*483*, *490*
2	20.50%	(15.1–27.0)	0.97	(0.62–1.53)		0.99	(0.63–1.56)		*306*, *303*
3–4	17.40%	(13.9–21.6)	0.8	(0.55–1.15)		0.79	(0.55–1.14)		*532*, *528*
5–9	16.00%	(13.1–19.2)	0.72	(0.50–1.02)		0.68	(0.48–0.97)		*865*, *900*
10+	16.40%	(14.3–18.7)	0.74	(0.53–1.02)		0.69	(0.49–0.96)		*1316*, *1361*
**Same sex experience, ever** ^**4**^					p = 0.8434			p = 0.8289	
No	17.40%	(15.9–19.0)	1	-		1	-		*3343*, *3436*
Yes	18.00%	(12.7–25.0)	1.04	(0.68–1.60)		1.05	(0.68–1.61)		*202*, *189*
**Number of sexual partners** ^**3**^ **, past year**					p = 0.723			p = 0.7831	
0	17.80%	(12.2–25.3)	1	-		1	-		*189*, *172*
1	17.70%	(15.8–19.7)	0.99	(0.62–1.58)		0.98	(0.61–1.56)		*2294*, *2581*
2	14.50%	(11.0–18.8)	0.78	(0.46–1.35)		0.84	(0.49–1.47)		*421*, *355*
3–4	18.20%	(13.7–23.7)	1.03	(0.59–1.79)		1.1	(0.63–1.93)		*354*, *287*
5+	17.80%	(12.9–24.1)	1	(0.55–1.81)		1.1	(0.60–2.01)		*251*, *193*
**Number of sexual partners** ^**3**^ **without a condom, past year**					p = 0.6247			p = 0.9503	
0	16.50%	(13.6–20.0)	1	-		1	-		*859*, *781*
1	17.80%	(15.8–19.9)	1.09	(0.83–1.43)		1.03	(0.78–1.35)		*2104*, *2372*
2+	15.80%	(12.4–20.0)	0.95	(0.65–1.38)		0.98	(0.67–1.41)		*513*, *410*

1 Unweighted, weighted denominators: all 16–44 year old men reporting at least one lifetime partner by the time of their interview for Natsal-3

2 Participants aged ≥17 years

3 Includes both opposite and same-sex partners

4 Same-sex experience involving genital contact

Circumcision was not associated with reporting previous diagnosis/es of any STI, or specifically chlamydia or genital warts in 16–44 year old men (Table 2). Results were similar in 16–74 year old men (data not shown). In unadjusted analyses, any HPV type was less likely to be detected in circumcised men as were HR-HPV and possible HR-HPV types (but not HPV types 6 and 11; Table 2). Circumcised men were also less likely to have *C*. *trachomatis*, but not *M*. *genitalium* detected (Table 2). After adjusting for key demographic and behavioural variables (Table 2), circumcised men were still less likely to have any HPV-type detected in their urine when compared with uncircumcised men (AOR 0.26, 95% CI 0.13–0.50). An association also remained for both HR-HPV (AOR 0.14, 95% CI 0.05–0.40) and possible HR-HPV (AOR 0.24, 95% CI 0.06–0.94) and for *C*. *trachomatis* (AOR 0.09, 95% CI 0.01–0.77). Unadjusted and adjusted ORs for the association between circumcision and *M*. *genitalium* differed substantially due to confounding by ethnicity.

**Table 2:** Variations in the reported diagnosis of, and detection of, current infection, of key STIs among sexually-experienced men aged 16–44 years in Britain’s third National Survey of Sexual Attitudes and Lifestyles (Natsal-3).

	Circumcised men		Uncircumcised men					
	%	(95%CI)	%	(95%CI)	OR	(95%CI)	AOR^1^	(95%CI)
***Reported diagnosis of*:**								
*Denom*. *(unwt*, *wt)* ^*2*^	*561*, *627*		*2969*, *2980*					
Any STI ^3^	13.7%	(10.8–17.2)	13.9%	(12.4–15.5)	0.98	(0.73–1.32)	1.05	(0.75–1.48)
Chlamydia infection	6.5%	(4.8–8.8)	6.2%	(5.3–7.2)	1.06	(0.73–1.52)	1.23	(0.81–1.86)
Genital warts	3.5%	(2.2–5.6)	3.9%	(3.1–4.9)	0.88	(0.51–1.51)	1.05	(0.60–1.86)
***Current infection with*:**								
*Denom*. *(unwt*, *wt)* ^*4*^	*284*, *403*		*1566*, *1821*					
HPV (any)	6.4%	(4.0–10.2)	18.6%	(16.1–21.3)	0.30	(0.18–0.51)	0.26	(0.13–0.50)
HR-HPV ^5^	2.3%	(1.0–4.9)	9.8%	(7.8–12.2)	0.21	(0.09–0.49)	0.14	(0.05–0.40)
Possible HR-HPV ^6^	0.6%	(0.2–2.0)	2.4%	(1.6–3.7)	0.25	(0.07–0.86)	0.24	(0.06–0.94)
HPV-6/11	0.7%	(0.2–2.5)	1.5%	(0.8–2.7)	0.48	(0.11–2.00)	0.58	(0.16–2.10)
*Chlamydia trachomatis*	0.1%	(0.0–0.7)	1.3%	(0.9–1.9)	0.08	(0.01–0.59)	0.09	(0.01–0.77)
*Mycoplasma genitalium*	1.9%	(0.7–5.1)	1.0%	(0.6–1.7)	1.90	(0.62–5.87)	0.61	(0.18–2.09)

1 ORs adjusted for age, ethnicity, same sex experience and number of partners in lifetime (for diagnosis outcomes) or number of partners without a condom in the past year (for biological outcomes)

2 Unweighted, weighted denominators: all 16–44 year old men reporting at least one lifetime partner by the time of their interview for Natsal-3

3 Defined as chlamydia, gonorrhoea, genital warts, syphilis, trichomonas, herpes, NSU/NGU, epididymo-orchitis, public lice/crabs or hepatitis

4 Unweighted, weighted denominators: 16–44 year old men with one or more lifetime partner who provided a urine sample

5 Positive for type(s) 16, 18, 31, 33, 35, 39, 45, 51, 52, 56, 58, 59 and/or 68

6 Positive for type(s) 26, 53, 66, 70, 73 and/or 82

## Discussion

Findings from this large British population-based survey show that male circumcision is strongly associated with lower detection of any HPV and HR-HPV in urine, even after adjusting for key demographic and behavioural variables. However, circumcision was not associated with self-reported previous STI diagnosis/es in this population.

The strengths of this study are that the initial Natsal-3 sample size is large, and probability sampling gives data representative of the British general population.[11] In contrast, studies included in the meta-analysis of the association between HPV and genital warts and circumcision were generally convenience samples with relatively small sample sizes.[8] In addition to using self-reports of STI diagnosis/es, which may be subject to reporting bias (despite using CASI to collect these data [11]), Natsal-3 used biological markers of STI, which have greater reliability.[17] However, as the population prevalence of some of the STIs considered was low (specifically HPV-6/11, *C*. *trachomatis* and *M*. *genitalium*),[5] statistical power was too low to detect modest differences in prevalence. Thus, the observed strong association between circumcision and lower detection of Chlamydia should be interpreted with caution since the 95%CIs around this are wide. It is also necessary to consider the method of sample collection. Urine tests for *C*. *trachomatis* and *M*. *genitalium* are regarded as the ‘gold standard’ in men, but urine is considered a suboptimum specimen for detection of HPV. Hernandez *et al* assessed HPV prevalence at different sites and found that it was highest in the penile shaft (52% at this site) compared to just 10% in urine,[18] suggesting that HPV prevalence may be underestimated in our study. Another study found no difference in prevalence of HR-HPV in urethral swabs when comparing swabs taken before and after circumcision.[19] Although detection bias remains a possible explanation for our findings if the presence of the foreskin means that urine may collect HPV coming from other sites (e.g. the glans/corona,[18,20,21] where prevalence is higher), in addition to the urethra. However, a systematic review and meta-analysis, which included data collected from a variety of anatomical sites, found results similar to our study and a stronger effect of circumcision at sites more proximal to the foreskin (urethra and glans/corona)[8] suggesting that the results presented here may not solely be due to differential detection.

The lack of association between male circumcision and self-reported previous STI diagnoses is in keeping with Natsal-2 findings,[22] and the meta-analysis of the association between circumcision and HPV,[8] which reported significantly lower odds of any HPV type (at any anatomical site) in circumcised men compared to uncircumcised men but no association with genital warts. Our data suggest an association between circumcision and lower odds of detection of *C*. *trachomatis*, consistent with some,[10] but not all other studies.[9] However, we did not find an association with reported diagnosis of Chlamydia. We are unable to compare our findings for HPV-6/11 or *M*. *genitalium* to the findings of other studies due to insufficient statistical power.

Data from a large national probability survey have shown that male circumcision is associated with lower odds of detecting oncogenic HPV types. Reduced prevalence of oncogenic HPV types among men may result in reduced transmission to sexual partners, with the consequent effect of reduced risk of cervical cancer. Castellasgue *et al*. observed that monogamous women whose partners had reported six or more sexual partners and were circumcised were at lower risk of cervical cancer than monogamous women whose partners were uncircumcised.[23] Currently the World Health Organisation advocates male circumcision for HIV prevention;[24] reduction in HPV acquisition would add to the overall health benefit resulting from the scale-up of male circumcision in countries with high prevalence of HIV. In such settings this reduction could be important due to the higher incidence of cervical cancer, particularly among HIV positive women, as well as other HPV related cancers in both men and women than in higher income countries,[25] and the low or absent HPV vaccination coverage. This present study shows that male circumcision is associated with lower odds of detection of HR-HPV types, meaning it may convey a long-term public health benefit. Future research should be directed towards determining whether male circumcision might contribute to reducing rates of cervical, penile, anal and oral cancers.
